# Transcriptomic Analysis Provides Insights into Flowering in Precocious-Fruiting *Amomum villosum* Lour.

**DOI:** 10.3390/plants15020198

**Published:** 2026-01-08

**Authors:** Yating Zhu, Shuang Li, Hongyou Zhao, Qianxia Li, Yanfang Wang, Chunyong Yang, Ge Li, Wenlin Zhang, Zhibin Guan, Lin Xiao, Yanqian Wang, Lixia Zhang

**Affiliations:** 1 Institute of Medicinal Plant Development, Chinese Academy of Medical Sciences & Peking Union Medical College, Beijing 100193, China; s2023009041@pumc.edu.cn; 2 Yunnan Key Laboratory of Southern Medicinal Utilization, Yunnan Branch of Institute of Medicinal Plant Development, Chinese Academy of Medical Sciences & Peking Union Medical College, Jinghong 666100, China; k20071129@163.com (S.L.); hyzhao@implad.ac.cn (H.Z.); 15925416204@163.com (Q.L.); 18088050877@163.com (Y.W.); ace928@126.com (C.Y.); lige19800221@163.com (G.L.); 18187706297@163.com (W.Z.); 13578155680@163.com (Z.G.); 13308811908@163.com (L.X.); 3 Yunnan Key Laboratory of Sustainable Utilization of Southern Medicine, Yunnan University of Traditional Chinese Medicine, Kunming 650500, China

**Keywords:** *Amomum villosum* Lour., precocious fruiting, flowering, spatiotemporal specificity, transcriptome sequencing

## Abstract

Precocious-fruiting *Amomum villosum* Lour. is characterized by early fruit set, rapid yield formation, and shortened economic return cycles, indicating strong cultivation potential. However, the molecular mechanisms underlying its flowering transition remain unclear. To elucidate the flowering mechanism of *A. villosum*, we used the Illumina NovaSeq X Plus platform to compare gene expression profiles in three tissues (Rhizomes, R; Stems, S; Leaves, L) during the vegetative stage and three tissues (Rhizomes and Inflorescences, R&I; Stems, S; Leaves, L) during the flowering stage of individual plants: VS-R vs. FS-R&I, VS-S vs. FS-S, and VS-L vs. FS-L. We obtained 52.5 Gb clean data and 789 million reads, and identified 2963 novel genes. The 3061 differentially expressed genes (DEGs, FDR ≤ 0.05 and |log2FC| ≥ 1) identified in the three comparison groups included six overlapping genes. The DEGs were enriched primarily in GO terms related to cellular process, metabolic process, binding, catalytic activity, and cellular anatomical entity, as well as multiple terms associated with development and reproduction. KEGG enrichment analysis revealed enrichment primarily in metabolic pathways, including global and overview maps, energy metabolism, and carbohydrate metabolism. Moreover, the most significantly enriched core pathways included metabolic pathways, photosynthesis, and carbon assimilation. Among all alternative splicing (AS) events, skipped exons (SEs) accounted for the largest proportion (59.5%), followed by retained introns (RI, 19.4%), alternative 3′ splice sites (A3SS, 10.7%), alternative 5′ splice sites (A5SS, 6.8%), and mutually exclusive exons (MXE, 3.6%). A preliminary set of 43 key DEGs was predicted, displaying spatiotemporal expression specificity and strong interactions among certain genes. Nine genes were further selected for RT-qPCR validation to confirm the reliability of the RNA-seq results. This study established a foundational framework for elucidating the flowering mechanism of precocious-fruiting *A. villosum*.

## 1. Introduction

**Figure 1:**
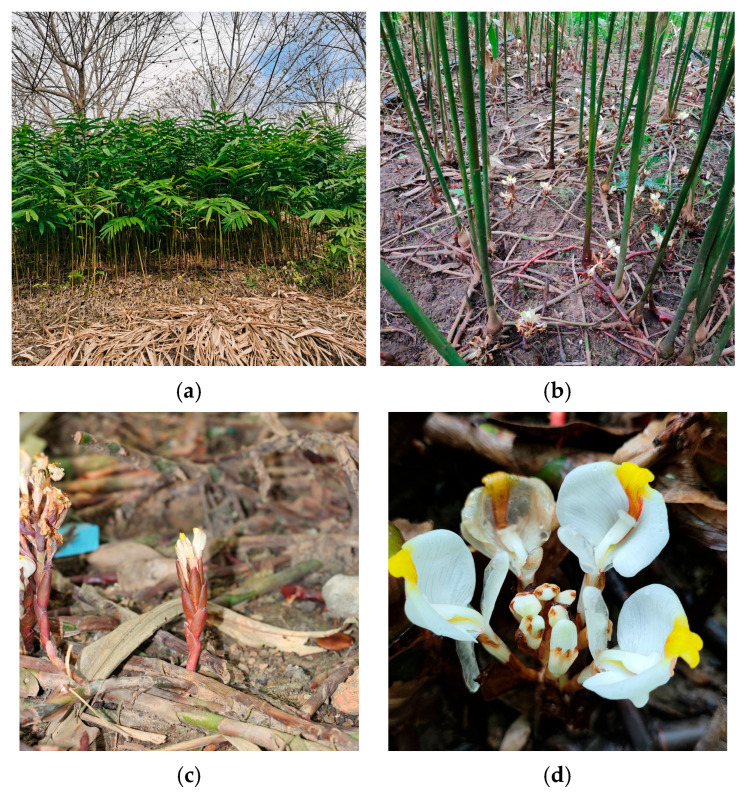
Growth environment and floral-related traits of *A. villosum*: (**a**) field growth environment and plant morphology of *A. villosum*; (**b**) overall flowering status of *A. villosum*; (**c**) flowers borne on prostrate rhizomes on the ground; (**d**) floral morphology of *A. villosum*.

*Amomum villosum* Lour. (*A. villosum*) is a perennial herbaceous plant belonging to the genus *Amomum* Roxb. in the Zingiberaceae family. It is one of the original plants in the traditional Chinese medicine “Sha Ren” (*Fructus Amomi*), and its mature fruits have pharmacological effects of resolving dampness and promoting appetite, warming the spleen to relieve diarrhea, regulating qi, and calming the fetus [1]. The plant grows to a height of 2.0–3.0 m and has both rhizomes and erect stems. The elongated rhizomes creep along the ground and have membranous brown scales on the nodes. The erect stems are scattered, unbranched, and swollen at the base, thereby forming a bulbous shape. The inflorescence of *A. villosum* is a spike, which arises from the rhizomes creeping along the ground. Flower buds emerge from the rhizomes from March to April, the inflorescences mature and open from May to June, and the plant enters the fruiting period from July to September [2]. In *A. villosum* flowers, the stigma of the pistil is higher than the anther of the stamen. As an entomophilous plant, *A. villosum* relies on insects for pollination. The florets bloom gradually from the bottom to the top. The blooming period of a single floret is generally approximately 1 day, and the time required for all florets on one inflorescence to finish blooming usually ranges from 3 to 9 days [3] (Figure 1d). *A. villosum* can propagate seedlings through two modes: sexual propagation by seeds and asexual propagation by division. Seedlings that are propagated asexually by division bloom and enter the reproductive growth stage 2–3 years after planting, whereas seedlings propagated sexually by seeds require 3–4 years to bloom [2]. We previously discovered a precocious fruiting phenomenon in *A. villosum*, in which flowering and fruiting occur during the first year after clonal propagation. This finding notably shortens the juvenile phase of *A. villosum*, thus providing a solid foundation for genetic improvement.

Rhizome-borne inflorescence represents a rare reproductive strategy in the plant kingdom. In addition to *A. villosum*, this characteristic is also manifested in the other two pharmacopoeial source species of Fructus Amomi: *Amomum villosum* var. *xanthioides* and *Amomum longiligulare* T. L. Wu. Furthermore, this trait is observed in several congeneric species within the genus *Amomum* Roxb., including *Amomum tsaoko*, *Amomum maximum* Roxb., and *Amomum hypoleucum* Thwaites. Despite its uniqueness, the molecular and physiological mechanisms underlying rhizome-borne flowering remain poorly understood, representing a significant research gap in plant reproductive biology.

Studies of the model plant *Arabidopsis thaliana*, and other species such as rice (*Oryza sativa* L.) [4,5,6,7], maize (*Zea mays* L.) [7,8], and *Brachypodium distachyon* [9], have demonstrated that flowering in plants is regulated primarily through six pathways: the photoperiod, vernalization, gibberellins, autonomous, thermosensitive, and age pathways. These six pathways synergistically interact, and the integration of internal and external signals ultimately leads to flowering initiation. For example, in the photoperiod pathway, genes exhibit spatiotemporal-specific expression. Typically, plants perceive light through various photoreceptors in their leaves. After light signals are received, they are transmitted to the circadian clock [10,11,12], which further conveys photoperiodic signals to *CONSTANS* (*CO*). Subsequently, *CO* regulates its downstream flowering integrators *FLOWERING LOCUS T* (*FT*) and *SUPPRESSOR OF OVEREXPRESSION OF CONSTANS 1* (*SOC1*) [13], thus promoting the transport of FT through the phloem from the light-sensing tissues (leaves) to the responsive tissues (shoot apical meristem) [14]. After *FT* reaches the shoot apex, it activates downstream floral meristem identity genes such as *FD*, *APETALA 1* (*AP1*), and *LEAFY* (*LFY*), thereby initiating the flowering process at the floral site [15]. This body of work demonstrates that flowering-related genes are expressed in various tissues throughout the plant, highlighting the necessity of conducting comparative analyses using different organs—such as rhizomes, stems, and leaves—in our study of *A. villosum*.

Transcriptome sequencing (RNA-seq) enables high sensitivity, throughput, and accuracy, and it has been widely applied in studies on plant growth, development, and secondary metabolism. This technology facilitates a comprehensive understanding of gene expression patterns and regulatory networks involved in plant growth, development, and other physiological processes. Transcriptomic studies on flowering mechanisms have achieved substantial progress in various plant species such as *Oryza sativa* L. [16,17,18], *Osmanthus fragrans* [19,20], and *Dendrobium devonianum* [21]. These studies have leveraged this technology to systematically identify and functionally validate key genes and core pathways regulating flowering time and floral organ development, thereby further refining the theoretical framework of the plant flowering regulatory network. Several RNA-seq studies have also been conducted in *A. villosum*, focusing mainly on terpenoid biosynthesis [22,23]. However, transcriptome-based investigations focusing specifically on flowering regulation in *A. villosum* remain scarce.

Previously, we compared the differences between precocious-fruiting and non-precocious-fruiting strains of *A. villosum* through RNA-seq. Numerous differentially expressed genes (DEGs) involved in plant hormone signal transduction, carbohydrate metabolism, polysaccharide degradation, and lipid metabolism pathways were identified to participate in the precocity process in *A. villosum* [24]. On the basis of these results, we further cloned and preliminarily verified the flowering genes *AvFD1* [25] and *AvGhd7* [26], whose expression in flowering tissues was significantly different from non-flowering tissues, thus demonstrating tissue-specific expression of flowering-associated genes in *A. villosum*.

However, the earlier study had three key limitations: (1) crude mixed-tissue sampling (e.g., whole above-ground parts) failed to distinguish gene expression in individual organs (rhizomes, stems, and leaves) and masked tissue-specific regulatory signals; (2) Lack of temporal resolution (only static precocious vs. non-precocious comparison) missed the vegetative-to-flowering transition and dynamic floral regulatory processes; (3) absence of a genomic reference forced de novo transcriptome assembly, leading to low gene annotation completeness and limited detection of AS events.

To address these, the current study optimized its design: (1) we used a clonally propagated, genetically uniform precocious cultivar, (2) focused on the flowering transition of precocious-fruiting *A. villosum*, and conducted RNA-seq on three key tissues–rhizomes (and inflorescences), stems, and leaves across two developmental stages: the vegetative stage and flowering stage. This sampling scheme was designed to establish a “space (tissue)-time (developmental stage)” framework for spatiotemporal-resolved analysis. Specifically, for the flowering stage (FS), we deliberately selected the period when flower buds and fully opened flowers coexist. This FS-specific sampling was intended to ensure that DEGs identified between the two developmental stages would not only include those involved in sustaining the late flowering program and maintaining continuous expression during early flowering initiation signal transduction but also genes associated with resource reallocation and tissue functional specialization throughout the flowering period; (3) adopted the newly released *A. villosum* reference genome [22] to improve gene annotation accuracy, DEG identification reliability, and AS detection sensitivity.

In this study, we performed RNA-seq on the rhizomes (and inflorescences), stems, and leaves of *A. villosum* precocious-fruiting individuals in the vegetative growth stage or flowering stage, to identify key genes and signaling pathways associated with flowering in precocious-fruiting plants. The results of this analysis are intended to provide a candidate gene pool and directional guidance for subsequent studies, lay a foundation for clarifying the flowering regulatory network of *A. villosum*, and further promote research on the genetic improvement of this species.

## 2. Results

### 2.1. RNA-Seq Data Analysis

Sequencing was performed on the Illumina NovaSeq X Plus platform. Across 18 samples, a total of 52.5 Gb of clean data and approximately 789 million reads were obtained. The total mapped reads ranged from 94.85% to 96.26%, the uniquely mapped reads ranged from 91.46% to 93.03%, the GC content ranged from 46.12% to 50.01%, and the percentage of Q30 bases exceeded 95.56% for all samples (Table 1). In addition, 2963 novel genes were identified (Supplementary Table S1). These results indicated that the RNA-seq data were of high quality and, therefore, were suitable for downstream analyses of differential gene expression and AS events.

### 2.2. DEG Analysis

**Figure 2:**
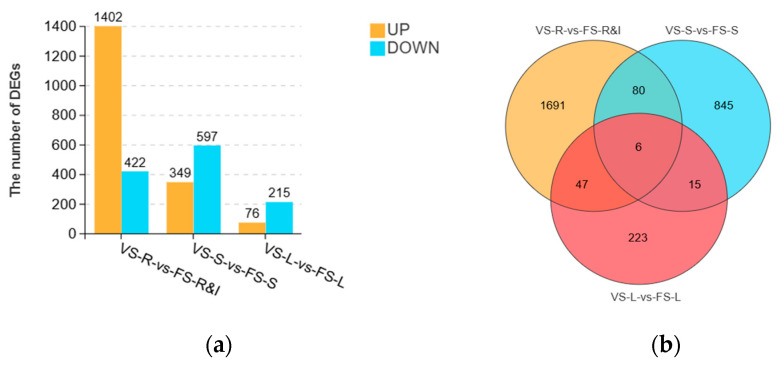
The statistical analysis of the number of DEGs in the three comparison groups (with biological replicates): (**a**) Bar chart showing numbers of upregulated (orange) and downregulated (blue) DEGs; (**b**) Venn diagram of DEGs across comparisons.

DEGs were screened according to the criteria of false discovery rate (FDR) ≤ 0.05 and |log2FC| ≥ 1. Analysis of the identified DEGs revealed 3061 DEGs across the three comparison groups, including six DEGs shared across all groups. In the VS-R vs. FS-R&I comparison, 1824 DEGs were detected, comprising 1402 significantly upregulated and 422 significantly downregulated genes, representing the largest number of DEGs among the three comparisons. In the VS-S vs. FS-S comparison, 946 DEGs were identified, comprising 349 upregulated and 597 downregulated genes. The fewest DEGs (*n* = 291) were found in the VS-L vs. FS-L comparison (Figure 2). These results indicate that gene expression in rhizomes and inflorescences, stems, and leaves undergoes substantial changes during the flowering stage of *A. villosum*.

### 2.3. Gene Ontology (GO) Functional Annotation and Enrichment Analysis of DEGs

**Figure 3:**
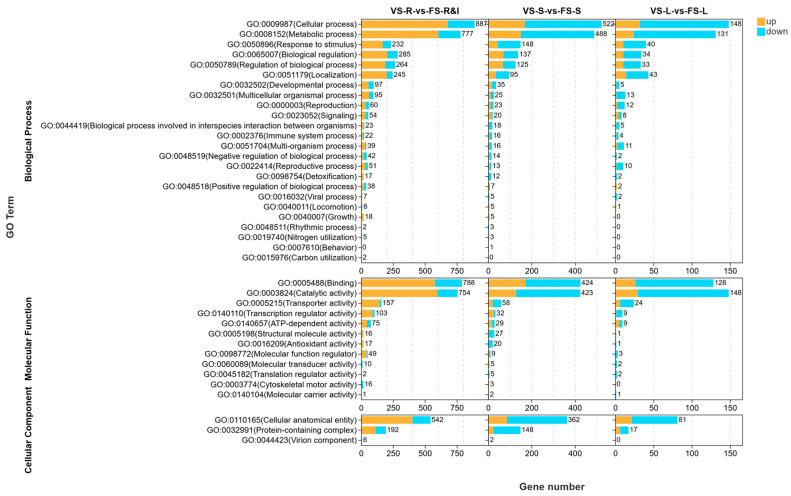
Classification of GO functional annotations for DEGs, with the upper part of the BP image, the middle part of the MF image, and the lower part of the CC image. The functional classification was conducted based on the results of DEG detection. Each major category has various subcategories at different levels, divided into 35 subtypes.

Figure 3 shows the GO annotation categories for the DEGs. The GO categories comprised three main domains: Biological Process (BP), Molecular Function (MF), and Cellular Component (CC). In the BP domain, the DEGs were enriched primarily in cellular process (GO:0009987), metabolic process (GO:0008152), response to stimulus (GO:0050896), biological regulation (GO:0065007), and regulation of biological process (GO:0050789). These pathways were represented by numerous DEGs across multiple comparisons. Additionally, response to stimulus (GO:0050896) might involve responses to environmental cues that, in turn, affect flowering time. Enrichment in developmental process (GO:0032502), reproduction (GO:0000003), and reproductive process (GO:0022414) indicated that genes associated with development and reproduction have important roles in flowering regulation. Notably, the VS-R vs. FS-R&I comparison revealed markedly more enriched genes than the other comparisons, thus suggesting that rhizome gene expression strongly influences flowering in *A. villosum*. In the MF domain, binding (GO:0005488) and catalytic activity (GO:0003824) were the two dominant categories, thus suggesting that many flowering-related genes encode enzymes and binding proteins involved in metabolism and signal recognition. Enrichment in transcription regulator activity (GO:0140110) and ATP-dependent activity (GO:0140657) additionally suggested links among flowering, transcriptional regulation, and energy metabolism. In the CC domain, the DEGs were enriched primarily in cellular anatomical entity (GO:0110165) and protein-containing complex (GO:0032991); consequently, flowering in precocious-fruiting *A. villosum* might involve the formation of developmental signaling complexes.

Overall, the GO enrichment analysis revealed marked differences before and after flowering, and among tissues in signal transduction, metabolic activity, transcriptional regulation, and reproductive development. The coordinated actions of these processes might systemically regulate the phase transition from vegetative to reproductive growth, thus laying an important foundation for elucidating the molecular regulatory network of flowering in precocious-fruiting *A. villosum*.

### 2.4. Kyoto Encyclopedia of Genes and Genomes (KEGG) Functional Annotation and Enrichment Analysis of DEGs

**Figure 4:**
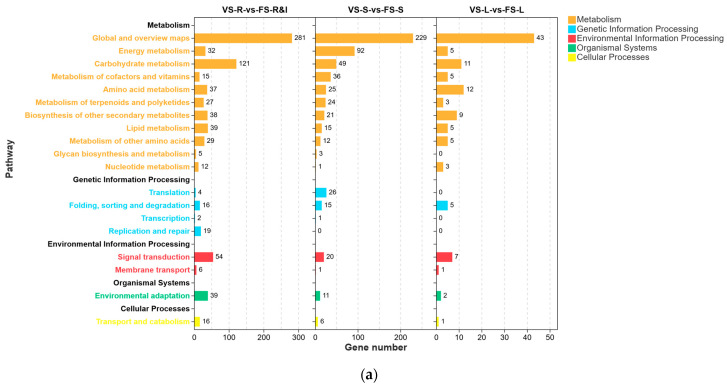

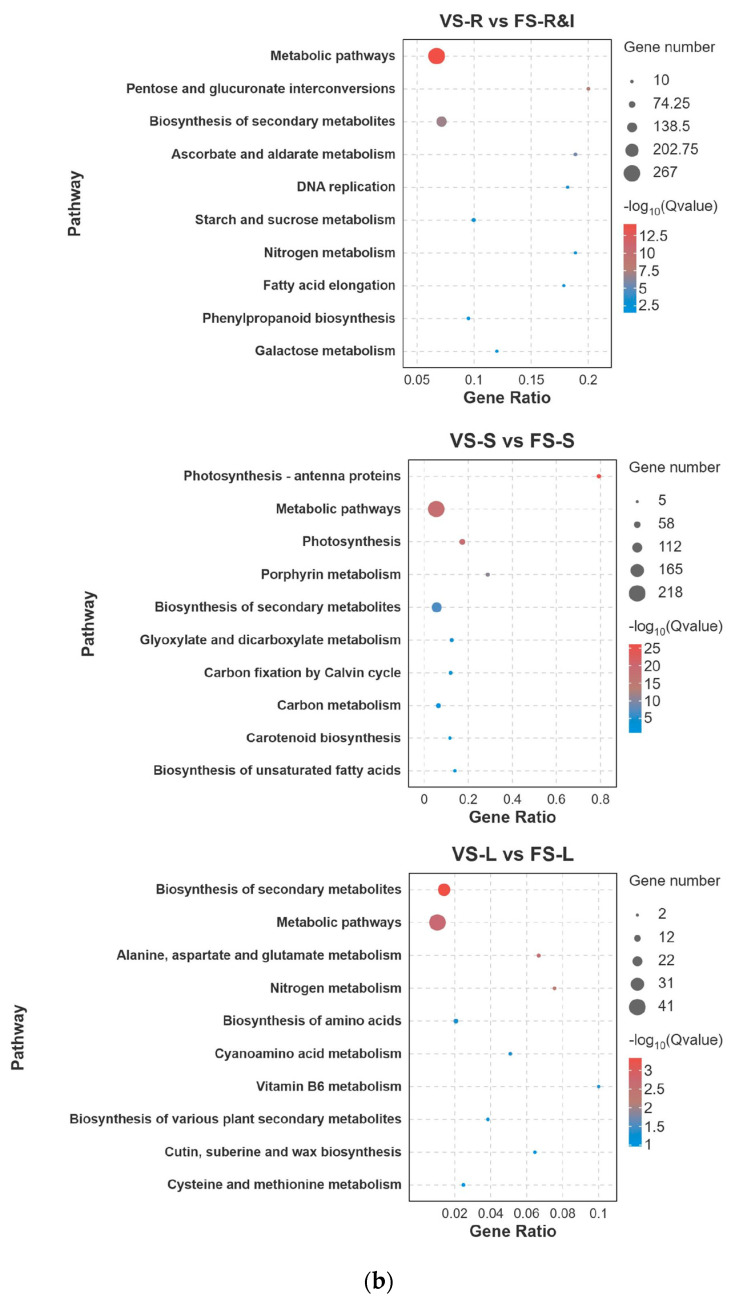
Results of KEGG enrichment for DEGs: (**a**) the KEGG pathway annotation classification results of DEGs; (**b**) a bubble chart of KEGG enrichment results for the three comparison groups.

The KEGG enrichment results (Figure 4) indicated five main branches: cellular processes, environmental information processing, genetic information processing, metabolism, and organismal systems. A total of 19 annotated pathways were identified across sub-branches.

Metabolism-associated pathways dominated across tissues and exhibited tissue specificity (Figure 4a). These pathways involved primarily global and overview maps, energy metabolism, carbohydrate metabolism, amino acid metabolism, and metabolism of terpenoids and polyketides. Therefore, metabolic activity appears to have important roles during the flowering of precocious-fruiting *A. villosum*.

We displayed the top ten enriched pathways ranked by FDR for each comparison (Figure 4b). In VS-R vs. FS-R&I, DEGs were significantly enriched in metabolic pathways (ko01100), pentose and glucuronate interconversions (ko00040), and ascorbate and aldarate metabolism (ko00053), among others. In VS-S vs. FS-S, the DEGs were concentrated in photosynthesis—antenna proteins (ko00196), metabolic pathways (ko01100), photosynthesis (ko00195), and carbon fixation by Calvin cycle (ko00710), representing photosynthesis and carbon assimilation pathways. In VS-L vs. FS-L, significant enrichment was observed in biosynthesis of secondary metabolites (ko01110), metabolic pathways (ko01100), and amino acid metabolism and synthesis/nitrogen metabolism (ko00250, ko01230, and ko00460). These results suggested that in precocious-fruiting plants, enhanced photosynthetic efficiency and carbon fixation might provide more energy and carbon skeletons for early reproductive development, thereby facilitating floral organ formation.

Overall, metabolic pathways, photosynthesis, and carbon assimilation were the highly enriched core pathways, thus providing important clues regarding the regulatory mechanisms of flowering in precocious-fruiting *A. villosum*.

### 2.5. Analysis of Alternative Splicing (AS) Events

**Figure 5:**
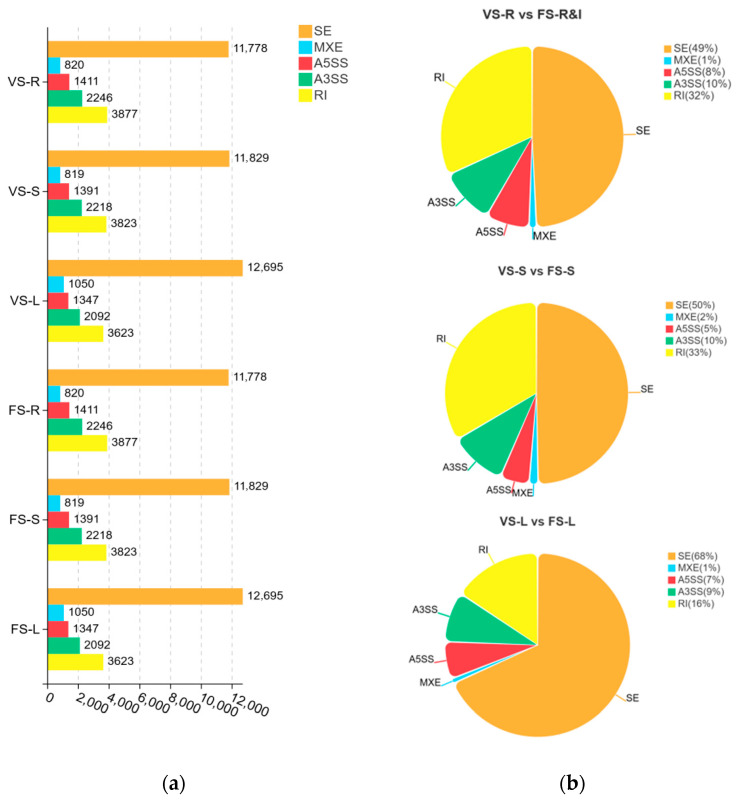
Systematic analysis results of AS events for different samples. There are five main types of AS events, including SE, MXE, 5SS, A3SS, and RI: (**a**) the statistical results of AS types for 6 groups of samples; (**b**) the statistical results of the differences in AS for 3 comparison groups in the form of a pie chart.

To explore the potential role of AS in precocity, we systematically analyzed AS events across samples (Figure 5).

Overall (Figure 5a), abundant AS events were detected in all samples, among which skipped exons (SE) accounted for the largest proportion (59.5%), and were followed by retained introns (RI, 19.4%), alternative 3′ splice sites (A3SS, 10.7%), and alternative 5′ splice sites (A5SS, 6.8%), whereas mutually exclusive exons (MXE) were the least abundant (3.6%). In differential AS comparisons (Figure 5b), SE and RI were the major differentially spliced gene (DSG) types across all three comparisons. We observed marked tissue-specific differences in AS types and proportions: in VS-R vs. FS-R&I, the proportion of SE DSGs comprised 47%, and RI DSGs comprised 34%; in VS-S vs. FS-S, the proportion of SE DSGs comprised 50%, and RI DSGs comprised 33%; and in VS-L vs. FS-L, the proportion of SE DSGs was 68%.

### 2.6. Analysis of Key Genes

To identify key candidate genes, we applied a multi-criteria screening strategy to the DEGs. The selection was based on the following sequential filters: (1) all candidate genes met the primary threshold of FDR ≤ 0.05 and |log2FC| ≥ 1; (2) functional relevance was prioritized, focusing on genes annotated with flowering-, development-, or reproduction-related keywords (e.g., “flower”, “floral”, “reproduction”) in GO terms or KEGG pathways; (3) broad tissue representation was considered, including genes showing expression changes across multiple comparison groups, notably the 6 DEGs shared by all three tissue comparisons; and (4) homology to known flowering regulators in model plants (e.g., *AP1, AP2/ERF, MADS-box, AGAMOUS (AG)*) was also evaluated. A gene was included in the final key gene set if it satisfied Criterion 1 and at least one of Criteria 2–4. Through this approach, we selected 43 high-confidence key DEGs for subsequent expression level and correlation analyses across tissues (Supplementary Tables S2 and S3).

**Figure 6:**
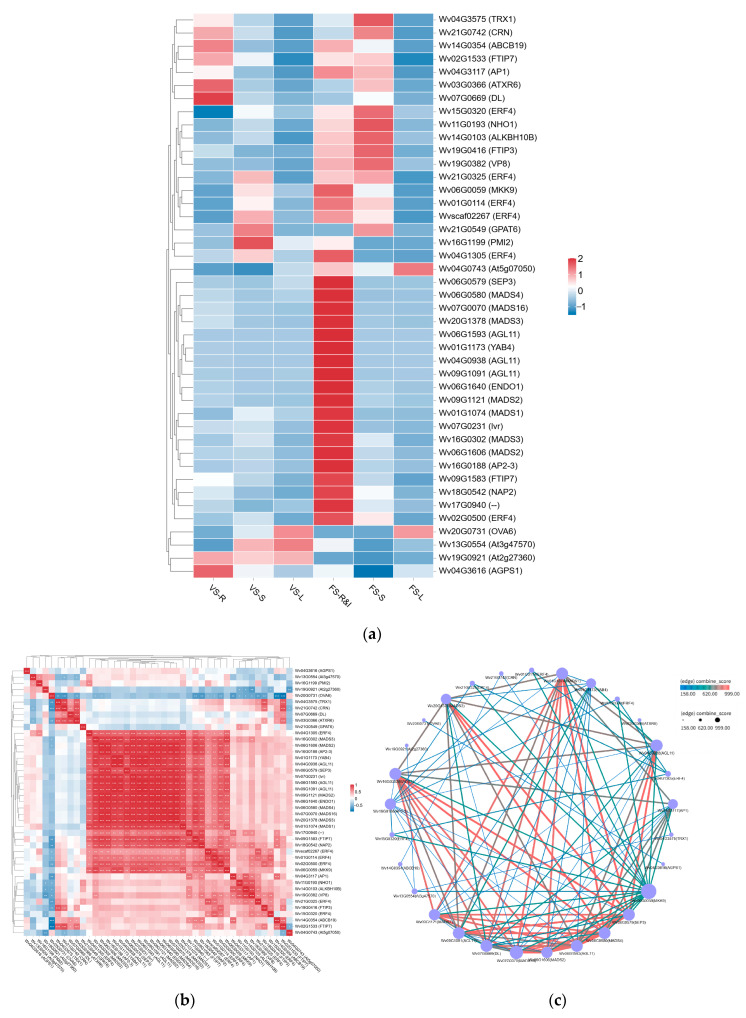
Analysis results of key genes: (**a**) heatmap showing the expression levels of key DEGs in different samples; (**b**) heatmap presenting the correlation analysis results of key differentially expressed genes, the asterisks (*, **, ***) denote statistical significance levels (*p*-value thresholds), with more asterisks indicating a higher level of significance. Specifically: * *p* < 0.05, ** *p* < 0.01, *** *p* < 0.001.; (**c**) PPI network analysis results of key DEGs, where the size of the points represents the connectivity of the genes, and the color of the lines indicates the combined score. The darker the color, the stronger the interaction relationship.

The results in Figure 6a show that the expression of these genes commonly exhibits specificity across both developmental stages and tissue types. Most genes showed higher expression in rhizome tissues, with 21 genes (48.8% of the total) being highly expressed specifically in FS-R&I. Furthermore, over half of the genes were highly expressed during FS overall. Cluster analysis revealed a clear grouping of samples by developmental stage and tissue type, indicating that these key genes may be involved in the coordinated regulation of tissue differentiation or developmental processes.

To uncover the potential regulatory relationships among key DEGs, we performed Pearson correlation analysis (Figure 6b). The heatmap indicated that most genes exhibited significant positive or negative correlations. Notably, we identified several highly correlated gene clusters: one cluster included 23 genes such as *Wv06G0059* and *Wv16G0302*; a second cluster comprised *Wv20G0731*, *Wv04G3575, Wv21G0742*, and *Wv07G0669*; and a third cluster included 7 genes like *Wv04G3117, Wv11G0193*, and *Wv15G0320*. These highly correlated co-expression modules suggest that their member genes may share similar biological functions or participate in the same signaling pathway, making them key targets for further investigation.

Protein–protein interaction network analysis revealed interaction relationships among the key proteins (Figure 6c). Our analysis revealed that Wv06G0579 (SEP3), Wv06G0580 (MADS4), and Wv07G0070 (MADS16) exhibited strong associations with other genes in the MADS-box family, with Combined Scores all above 900. Furthermore, genes such as Wv04G0938, Wv06G0059, and Wv01G1173 showed multiple interaction connections. In total, we identified 76 gene pairs with a Combined Score greater than 500 (Supplementary Table S4).

### 2.7. RT-qPCR Analysis of DEGs

**Figure 7:**
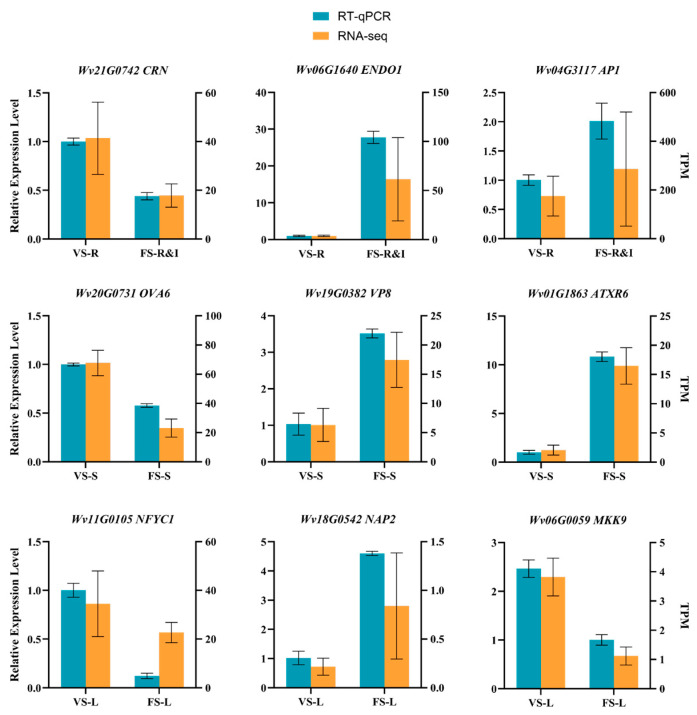
Verification of RNA-seq results by RT-qPCR.

Nine DEGs were selected randomly from the aforementioned key genes for RT-qPCR validation (relative expression and transcriptomic results in Figure 7). The expression trends of these nine genes were consistent with those observed in the RNA-seq data, confirming the reliability of the transcriptome data.

## 3. Discussion

This study differs substantially from previous transcriptomic investigations of precocious fruiting in *A. villosum* in terms of experimental design and analytical resolution. It represents an evolution in both material selection and research strategy, addressing several key limitations of earlier work. Despite differences in annotation systems that preclude direct gene-level comparison, the two studies share several core biological conclusions. GO enrichment analyses shared several major categories (e.g., cellular process, metabolic process), and both highlighted the global and overview maps as the KEGG pathway containing the most DEGs, with carbohydrate metabolism also commonly enriched, supporting the view of flowering as an energy-intensive process [24]. Future plans include re-annotating the prior de novo transcriptomic data with the unified *A. villosum* reference genome to integrate both datasets, facilitating screening of core candidate genes differentially expressed across “strain divergence” and “spatiotemporal dynamics” for a more systematic understanding of the molecular regulatory network underlying *A. villosum* precocious flowering.

Gene symbols were obtained by BLAST-searching (https://www.uniprot.org/blast, accessed on 25 July 2025) sequences against the SWISS-PROT database, and preliminary functional descriptions were performed using the Non-Redundant Protein Sequence Database. The functional discussions presented herein are based on these annotation results; however, definitive functional identification remains subject to in-depth experimental research. Among the 43 core DEGs identified, several belong to the MADS-box family and the *AP2/ERF* transcription factor family. These genes show a high degree of correlation with well-established flowering regulatory networks in model plants.

The MADS-box transcription factor family is extensively involved in various plant developmental processes, including floral organ formation, root development, and seed and fruit development [27]. In flower development, this family acts as a key executor for determining floral organ identity and regulating flowering time, playing crucial roles in flowering initiation, floral meristem establishment, organ differentiation, and morphogenesis [28]. The expression dynamics and interaction networks of these genes collectively constitute the complex flowering mechanism from transcriptional regulation to the activation of developmental programs.

In this study, the gene *Wv04G3117* was annotated as an *AP1* homolog. *AP1* belongs to the FRUITFULL (FUL) subfamily of the MADS-box family. It participates in floral meristem formation, inflorescence architecture determination, and petal and sepal differentiation, serving as a critical node in flowering initiation and floral organ specification. In *Arabidopsis*, *AP1* is activated by the FT-FD complex and then initiates the floral organ development program in the shoot apical meristem, thereby facilitating the transmission of flowering signals [15]. Here, the expression level of *Wv04G3117* showed significant changes in rhizome and stem tissues during FS compared to VS. This finding aligns with previous reports of high *AvFD1* expression in flowering tissues [25], suggesting the potential involvement of a coordinated “root–stem” AP1-FD module in flowering regulation in *A. villosum*. Furthermore, the FTIP7 (FT-INTERACTING PROTEIN *7*) homologs (*Wv02G1533*, *Wv19G0416*) screened in this study may participate in this regulatory pathway. In established flowering models, FTIP functions as a transport protein whose core role is to mediate the long-distance movement of FT protein, ensuring the inter-tissue transmission of flowering signals. These results collectively suggest that the regulatory module composed of AP1-FD-FTIP may be one of the key pathways for flowering initiation in *A. villosum* [29,30].

*SEPALLATA3* (*SEP3*) is a class of floral organ accessory factors within the MADS-box family. Its expression is typically concentrated in whorls 2 and 3 of floral organs, where it regulates floral organ development by activating other genes. Takato et al. [31] found that *SEP3* is involved in the FPF1-regulated early flowering process in *Arabidopsis*. In pepper, *CaSEP3* interacts with *CaYABBY5* to regulate floral organ determinacy and fruit morphogenesis [32]. This study identified an *SEP3* homolog (*Wv06G0579*), which showed the highest expression in FS-R&I and was barely detectable in stems and leaves. PPI analysis indicated that this gene interacts with 13 key genes, 9 of which had combined scores above 900, suggesting it is a candidate gene worthy of focused investigation.

Homologs of another key gene, *AGL11* (*Wv04G0938*, *Wv09G1091*), were also identified. *AGL11* has traditionally been considered primarily responsible for regulating ovule and seed development. However, recent studies indicate its involvement in flowering regulation. For instance, in quinoa, *CqSTK* (an *AGL11* homolog) influences flowering time [33]; in *Zanthoxylum armatum*, *ZaNAC93* may cooperate with *AGL11* and other genes in floral induction, fruit growth, and trichome initiation [34]; and in sunflower, *HaAGL11* plays a significant regulatory role in ovary and fruit development during late flowering stages [35]. In this study, *AGL11* was specifically highly expressed in rhizome and inflorescence tissues during the FS. PPI analysis revealed a high degree of association between *AGL11* and the aforementioned *SEP3* homolog (*Wv06G0579*), indicating their potential cooperative role in flowering regulation.

The AP2/ERF transcription factor family participates in multiple developmental processes such as seed development, floral organ formation, and fruit morphogenesis by integrating hormone and environmental signaling networks. The gene *Wv16G0188* was annotated as an *AP2* homolog. In *Arabidopsis*, *AP2* possesses a dual function: it represses *FT* expression to delay flowering during the vegetative growth phase while maintaining sepal and petal identity through interaction with *AG* during the reproductive phase [36]. In this study, *Wv16G0188* was significantly upregulated in FS-R&I with a TPM of 76.12. PPI analysis showed strong interactions between this *AP2* homolog and several *ERF4* homologs (*Wv01G0114*, *Wv02G0500*, *Wv15G0320*, and *Wv21G0325*), as well as high interaction with several *MYB73* homologs (*Wv21G1309*, *Wv24G1623*, *Wvscaf01043*, *Wv15G1172*, and *Wv02G0229*). This suggests that AP2/ERF and MYB transcription factors may jointly constitute an important component of the flowering regulatory network in *A. villosum*.

In plants, AS extensively contributes to the regulation of key biological processes, including flowering time control, hormone signaling, and photoperiodic responses. Previous studies have demonstrated that core flowering regulatory genes such as *FT*, *CO*, and *FLOWERING LOCUS C (FLC)* can generate distinct splice isoforms that precisely modulate the timing of floral transition [37]. *FLOWERING LOCUS M* (*FLM*), also known as *MADS AFFECTING FLOWERING 1 (MAF1*), is a MADS-box transcription factor within the *FLC* family whose AS is regulated by ambient temperature. At low temperatures, the predominant splice variant is FLM-β, which functions as a floral repressor, whereas at elevated temperatures the dominant isoform switches to FLM-δ, promoting flowering. Both major isoforms interact with *SHORT VEGETATIVE PHASE* (*SVP*) proteins to form complexes that regulate *FT* expression and hence flowering time [38,39]. In this study, extensive AS events were also detected among DEGs in *A. villosum*; however, their biological functions remain unclear. Whether these AS events contribute to flowering regulation in *A. villosum*, whether they respond to environmental cues (e.g., temperature and light) through mechanisms analogous to *FLM*, and whether they are functionally linked to transcription factor families such as MADS-box and AP2/ERF, will require rigorous functional validation experiments. This is also a potential future research direction for us.

This study has certain limitations, which are mainly reflected in the following two aspects. Corresponding improvements will be made in subsequent research.

First, sampling intervals were relatively broad. Floral development in higher plants is a continuous regulatory process involving three core stages: floral induction, floral evocation, and floral development, with distinct regulatory networks at each stage [40]. However, this study only sampled two time points, failing to fully cover this continuous process. Notably, this may omit the expression patterns of key flowering initiation genes: such genes are often transiently activated during the early vegetative-to-reproductive transition and return to basal levels by the study-defined FS, leading to ineffective capture of their regulatory roles. To address this, subsequent studies will improve sampling time resolution by adding nodes during the VS-FS transition (e.g., bud differentiation, flower bud emergence) and supplementing late FS (early fruit stage) samples. Constructing continuous time-series transcriptome data will enable a systematic analysis of transcriptional dynamics during *A. villosum* flowering.

Second, the investigation into DEGs and AS events remains preliminary. For DEGs, only preliminary expression analysis of key candidates was performed—key regulators may be missed, necessitating more in-depth, refined analysis. Notably, the study is limited to transcriptome-level prediction: the specific functions of these genes must be validated via experiments like gene cloning and functional assays. For AS events, only basic typing and identification were completed, with the association between AS and *A. villosum* flowering traits remaining unclear. To clarify AS’s regulatory role in *A. villosum* early fruiting and flowering, two follow-up steps are needed: (1) validate the biological functions of key AS events via experiments like CRISPR/Cas9 editing and heterologous transformation; (2) establish an integrated DEG-AS analysis system to explore their synergistic regulation in floral induction. This will offer a more comprehensive molecular foundation for advancing *A. villosum*’s floral regulatory network.

## 4. Materials and Methods

### 4.1. Experimental Materials

We selected precocious-fruiting seedlings of the cultivar “Yunsha No.8” derived from the same batch of division of *A. villosum* and transplanted them to an *A. villosum* plantation in Mengla County, Xishuangbanna Prefecture, China (21°29′ N, 101°55′ E). They went through division, and the newly propagated plants flowered in the first year after transplantation. After three consecutive years of observation after transplantation, individuals that flowered early each year were identified as precocious-fruiting plants. In this study, we selected precocious-fruiting individuals that were divided and transplanted in the first year and showed good growth. Plant growth status was systematically monitored, and rhizomes, stems, and leaf tissues were collected at the vegetative stage (VS) and flowering stage (FS) according to predefined sampling criteria. Sampling criteria were as follows: Leaves(L): selected as “the second fully expanded young leaf counted from the top of the plant”; Stems(S): selected as “the stem segment 5–10 cm above the underground corm”; Rhizomes(R): for the VS, “the middle segment of above-ground rhizomes” was sampled; Rhizomes and Inflorescences(R&I): for the FS, “floral organs and the rhizome tissues where they are attached” were sampled. Each tissue was analyzed in three biological replicates denoted VS-R-1, VS-R-2, VS-R-3, VS-S-1, VS-S-2, VS-S-3, VS-L-1, VS-L-2, VS-L-3, FS-R&I-1, FS-R&I-2, FS-R&I-3, FS-S-1, FS-S-2, FS-S-3, FS-L-1, FS-L-2, and FS-L-3. Samples were immediately frozen in liquid nitrogen and stored at −80 °C to preserve their biological characteristics and relevant metabolic activity, and enable reliable downstream assays and analyses.

### 4.2. Experimental Design

#### 4.2.1. RNA Extraction and Sequencing

Total RNA was extracted from *A. villosum* tissues with an RNAprep Pure Polysaccharide and Polyphenol Plant Total RNA Extraction Kit (Tiangen Biotech Co., Ltd., Beijing, China). After passing concentration and integrity checks, samples were submitted to BGI-Shenzhen for sequencing on the Illumina NovaSeq X Plus platform and cDNA library construction.

#### 4.2.2. Quality Control, Read Mapping, and Transcript Assembly

Raw sequencing reads were quality-filtered using fastp (v0.18.0). Ribosomal RNA reads were removed by alignment to an rRNA database using Bowtie2 (v2.2.8). Clean reads were subsequently aligned to the *A. villosum* reference genome [22] (NCBI BioProject: PRJNA910288) using HISAT2 (v2.1.0) with default parameters. Transcript assembly and reconstruction were performed with StringTie (v1.3.1). Transcripts detected in this study but absent from the reference annotation were defined as novel genes.

#### 4.2.3. Gene Expression Quantification and Differential Expression Analysis

Gene expression levels were quantified as raw read counts and transcripts per kilobase of exon per million mapped reads (TPM) using RSEM (v1.2.19). Differential expression analysis was conducted using DESeq2 (v1.20.0). Genes with FDR ≤ 0.05 and |log2FC| ≥ 1 were considered significantly DEGs.

#### 4.2.4. Functional Annotation and Enrichment Analysis

GO enrichment analysis of DEGs was performed using the GO database (http://www.geneontology.org/, accessed on 19 July 2025), and significantly enriched GO terms were identified based on hypergeometric tests with Bonferroni correction (Q ≤ 0.05). KEGG pathway enrichment analysis was conducted using the KEGG database (https://www.genome.jp/kegg/, accessed on 19 July 2025), and pathways with Q ≤ 0.05 after multiple-testing correction were considered significantly enriched.

#### 4.2.5. AS Analysis

AS is an important gene regulatory mechanism in eukaryotes. We used rMATS (v4.0.1) to detect AS events and performed differential AS analysis by analyzing these biological replicates.

#### 4.2.6. Validation of DEGs Using RT-qPCR

Nine key DEGs (three each from rhizomes, stems, and leaves) were randomly selected from the sequencing results for RT-qPCR validation. This random selection was intended to provide an unbiased validation of the RNA-seq results, ensuring that the reliability assessment was representative and not influenced by subjective selection. Specific primers were designed with Primer Premier (v6.0) and synthesized by Beijing Liuhe Huada Gene Technology Co., Ltd. (Beijing, China) (primer sequences in Table 2). The template for RT-qPCR was cDNA synthesized by reverse transcription from the remaining RNA samples after transcriptome sequencing. Reactions were conducted with TaKaRa TB Green Premix Ex Taq II (TaKaRa, Kusatsu, Shiga, Japan) in a CFX96 Fluorescence Quantitative PCR Instrument (Bio-Rad, Hercules, CA, USA). Each 25 µL reaction contained 12.5 µL TB Green Premix Ex Taq II, 1 µL each of forward and reverse primers, 2 µL cDNA, and 8.5 µL ddH2O. A two-step amplification protocol was used, with 95 °C pre-denaturation for 30 s, followed by 95 °C for 5 s and 60 °C for 30 s for 40 cycles. Relative gene expression levels were calculated using the 2^−ΔΔCt^ method, with actin serving as the internal reference gene. Each sample was analyzed in three technical replicates.

## 5. Conclusions

Herein, we performed RNA-seq on the rhizomes (and inflorescences), stems, and leaves of precocious-fruiting *A. villosum* during the vegetative and flowering stages; analyzed DEGs and AS across various growth stages and tissues; and conducted functional annotation and enrichment analyses with the GO and KEGG databases to identify key pathways and predict critical genes. From 52.5 Gb clean data and 789 million reads, we identified 2963 novel genes, thereby enriching the genomic content of *A. villosum*. Moreover, we identified 3061 DEGs across the three comparison groups, including 1827 upregulated and 1234 downregulated DEGs. Six DEGs were identified in all three comparison groups. GO functional annotation revealed that the DEGs were enriched predominantly in GO terms such as cellular process, metabolic process, binding, catalytic activity, and cellular anatomical entity, and were also enriched in multiple GO terms related to development and reproduction. KEGG enrichment analysis revealed that metabolic pathways predominated among pathways and exhibited tissue-specific patterns. The metabolic pathways involved multiple biological processes, primarily global and overview maps, energy metabolism, and carbohydrate metabolism. The KEGG enrichment results indicated that metabolic pathways, photosynthesis, and carbon assimilation were the core highly enriched pathways. SE events were found to account for the largest proportion (59.5%) of AS in all samples, and were followed by RI (19.4%), A3SS (10.7%), A5SS (6.8%), and MXE (3.6%). A preliminary analysis identified 43 key DEGs, which exhibited spatiotemporal expression specificity and strong interactions among certain genes. Nine genes associated with flowering pathways were selected for RT-qPCR validation, which confirmed the RNA-seq results.

The core objective of this study was to construct a global transcriptional landscape associated with flowering in *A. villosum* by comparing transcriptomic differences across multiple tissues between the vegetative and flowering stages. In general, this study lays a foundation for dissecting the flowering regulatory mechanism of precocious-fruiting *A. villosum*. From a broader biological perspective, this study provides valuable insights into the flowering mechanisms of rhizomatous plants and asexually propagated plants. Compared with model plants, rhizomatous plants exhibit distinct specificities in flowering regulation, resource allocation, and organ synergy. However, the underlying molecular mechanisms have long been underexplored. Our findings offer preliminary evidence to help address this research gap. In terms of application value, the flowering time and flowering stability of *A. villosum* directly affect its fruit set rate and yield, which are among the key factors constraining its industrial development. The key flowering-related genes and regulatory pathways identified in this study offer potential targets and a theoretical basis for improving *A. villosum* varieties, stabilizing yield, and enhancing economic benefits through molecular breeding or cultivation regulation strategies.

It should be noted that this study remains a preliminary exploration of the transcriptional regulatory mechanism underlying flowering in *A. villosum*. Future research will focus on three main directions: (1) more fine-scale time-series sampling (e.g., covering key nodes such as pre-flowering initiation, bud formation, and full bloom); (2) functional verification of key candidate genes (e.g., via gene cloning, overexpression, or knockout approaches); and (3) integrated analysis of multi-omics data (transcriptomics, metabolomics, proteomics). Through these efforts, we aim to further dissect the core regulatory factors and their molecular networks governing precocious flowering in *A. villosum*, thereby providing more solid theoretical support for the genetic improvement and sustainable industrial development of *A. villosum*.

## Figures and Tables

**Table 1:** Evaluation of statistical results of RNA-seq data.

Sample	RawData (bp)	Clean Data (%)	Total Reads	Total Mapped Reads (%)	Unique Mapped Reads (%)	Multiple Mapped Reads (%)	Q30 (%)	GC (%)
VS-R-1	5,932,565,700	99.44	39,230,710	95.88	92.46	3.42	96.39	46.65
VS-R-2	6,232,267,200	99.43	41,146,432	95.39	91.79	3.60	95.65	46.65
VS-R-3	5,890,706,100	99.55	38,933,528	95.52	91.94	3.57	96.00	46.62
VS-S-1	5,703,252,900	99.75	37,774,236	96.12	92.56	3.56	97.73	46.48
VS-S-2	5,826,044,100	99.76	38,516,396	96.01	92.21	3.80	97.65	46.93
VS-S-3	5,770,274,400	99.52	38,185,776	96.06	92.57	3.49	97.59	46.76
VS-L-1	8,078,006,700	99.66	53,087,034	96.08	92.36	3.72	97.63	48.74
VS-L-2	6,720,999,000	99.57	44,158,670	96.15	92.22	3.93	97.48	48.81
VS-L-3	5,737,040,700	99.55	37,698,100	96.08	92.11	3.97	97.45	48.61
FS-R&I-1	6,349,645,800	99.66	42,101,572	95.56	92.22	3.34	96.27	46.88
FS-R&I-2	6,559,195,800	99.58	43,448,684	94.85	91.46	3.38	96.88	46.56
FS-R&I-3	7,291,182,900	99.71	48,378,628	95.75	92.40	3.35	96.82	46.67
FS-S-1	7,613,872,800	99.60	50,441,106	96.26	93.03	3.23	97.35	46.36
FS-S-2	6,998,250,600	99.71	46,475,966	96.23	93.00	3.22	97.29	46.68
FS-S-3	5,546,480,100	99.65	36,775,402	96.03	92.86	3.16	97.57	46.12
FS-L-1	7,438,798,200	99.66	42,101,572	95.56	92.22	3.34	96.27	46.88
FS-L-2	7,969,726,800	99.58	43,448,684	94.85	91.46	3.38	96.88	46.56
FS-L-3	7,792,803,600	99.71	48,378,628	95.75	92.40	3.35	96.82	46.67

**Table 2:** Primers for RT-qPCR validation.

Gene Name	Forward Primer Sequences (5′-3′)	Reverse Primer Sequences (5′-3′)
*Wv11G0105*	GCTCCGTGTAGCTCAGCAGAT	GTAGCACCTCAGCACCACCTTC
*Wv19G0382*	TGTCATTCTCGGCAACCACAGA	CATAACGGCGTGCGACATCAAG
*Wv01G1863*	GCAACATCGCCCGCTTCATCA	GCCGCTCGCCCTTCGTTATATC
*Wv20G0731*	TCTCATGTCCTCCCTCCCTCAC	GCGGACTGCGATACCTCTTCTT
*Wv18G0542*	CCAGACCAAGCAACACCTATCG	CTGGTTCCTGGTGGCTCTGTT
*Wv06G1640*	CCAGACGAAGCCTGCACCTT	GCTGCGAAGTGAAGTTGCTGAT
*Wv21G0742*	GATGATCCTCGGCGTGCTTCTC	GCAGATGCCTGATCCACCTTCC
*Wv06G0059*	CTGATGGTGGCGATCTGCTTGG	ACCGCTTCCCGCTTTCCTTGT
*Wv04G3117*	TGCTACCAAGGAACGGGAGGAG	GAGCATCCACGGAGGCAACAAG
*Actin*	CGCATTGACGACCTCCAGTG	TCTTCACCGCATGTGACAATCC

## Data Availability

Data are contained within this article or Supplementary Materials.

## Supplementary Materials

The following supporting information can be downloaded at: https://www.mdpi.com/article/10.3390/plants15020198/s1, Table S1: Novel Genes; Table S2: Information on the pathways directly related to flowering and the DEGs on the overall pathway; Table S3: Key genes information; Table S4: PPI results.

## Abbreviations

The following abbreviations are used in this manuscript:

*A. villosum*	*Amomum villosum* Lour.
R	Rhizomes
R&I	Rhizomes and Inflorescences
S	Stems
L	Leaves
VS	Vegetative Stage
FS	Flowering Stage
DEGs	Differentially Expressed Genes
AS	Alternative Splicing
GO	Gene Ontology
KEGG	Kyoto Encyclopedia of Genes and Genomes
BP	Biological Process
MF	Molecular Function
CC	Cellular Component
SE	Skipped Exon
RI	Retained Intron
A3SS	Alternative 3′ Splice Site
A5SS	Alternative 5′ Splice Site
MXE	Mutually Exclusive Exons
RNA-seq	Transcriptome sequencing
DSG	Differentially Spliced Gene
*CO*	*CONSTANS*
*FT*	*FLOWERING LOCUS T*
*SOC1*	*SUPPRESSOR OF OVEREXPRESSION OF CONSTANS 1*
*AP1/2*	*APETALA 1/2*
*LFY*	*LEAFY*
PPI	Protein–Protein Interaction
FUL	FRUITFULL
*AG*	*AGAMOUS*
*AGL11*	*AGAMOUS-LIKE 11*
ERF	Ethylene Response Factor
*FLM*	*FLOWERING LOCUS M*
*FLC*	*FLOWERING LOCUS C*
*FPF1*	*FLOWERING PROMOTING FACTOR 1*
*FTIP7*	*FT-INTERACTING PROTEIN 7*
*MAF1*	*MADS AFFECTING FLOWERING 1*
*SEP3*	*SEPALLATA3*
*SVP*	*SHORT VEGETATIVE PHASE*
TPM	Transcripts Per Million

## References

[1] Chinese Pharmacopoeia Commission (2020). Pharmacopoeia of the People’s Republic of China.

[2] Lin M., Tian H. (2015). Review of sexual reproduction characteristics of *Amomum villosum*. Subtrop. Plant Sci..

[3] Peng J., Li R., Li G., Wang Y. (2011). Study on the flowering dynamics, pollen viability and stigma receptivity of *Amomum villosum*. J. Yunnan Univ. Tradit. Chin. Med..

[4] Su Y., Yi Y., Ge S., Wang Z., Wei Z., Liu X., Zhang C., Wang H., Qian Y., Yu B. (2025). Circular RNAs derived from *MIR156D* promote rice heading by repressing transcription elongation of *pri-miR156d* through R-loop formation. Nat. Plants.

[5] Li C., Liu X.J., Yan Y., Alam M.S., Liu Z., Yang Z.K., Tao R., Yue E., Duan M., Xu J.H. (2022). *OsLHY* is involved in regulating flowering through the *Hd1*- and *Ehd1*-mediated pathways in rice (*Oryza sativa* L.). Plant Sci..

[6] Zhang K., Chen C., Li X., Yu J., Xu R., Li X., Wang P., Miao J., Tan W., Gong Z. (2025). *OsbZIP40* and *OsbZIP12* interact with *OsMFT1* to repress *Ehd1* expression and delay flowering in rice. Crop J..

[7] Yang J., Xu G., Zhang M., Xue W., Wu J., Li Y., Song G., Liu Y., Chen Y., Kong D. (2025). Dual role of *Glossy15* in regulating flowering by modulating gibberellins and floral organ gene expression in maize. New Phytol..

[8] Zicola J., Weber B., Tu X., Bader R., Zisis D., Aesaert S., Salvi S., Krajewski P., Van Lijsebettens M., Li C. (2025). *Vegetative to generative1 (Vgt1)* is an enhancer affecting flowering time and jasmonate signaling in maize by promoting the expression of *Zea mays Related to APETALA 2.7*. Plant Physiol..

[9] Zeng S., Qin Z. (2025). The FKF1–ELF3–PRC2 module regulates flowering time in response to light in temperate grasses. Proc. Natl. Acad. Sci. USA.

[10] Martínez-García J.F., Huq E., Quail P.H. (2000). Direct targeting of light signals to a promoter element-bound transcription factor. Science.

[11] Hicks K.A., Millar A.J., Carré I.A., Somers D.E., Straume M., Meeks-Wagner D.R., Kay S.A. (1996). Conditional circadian dysfunction of the *Arabidopsis* early-flowering 3 mutant. Science.

[12] Hall A., Bastow R.M., Davis S.J., Hanano S., McWatters H.G., Hibberd V., Doyle M.R., Sung S., Halliday K.J., Amasino R.M. (2003). The *TIME FOR COFFEE* gene maintains the amplitude and timing of *Arabidopsis* circadian clocks. Plant Cell.

[13] Imaizumi T., Kay S.A. (2006). Photoperiodic control of flowering: Not only by coincidence. Trends Plant Sci..

[14] Shim J.S., Kubota A., Imaizumi T. (2017). Circadian clock and photoperiodic flowering in *Arabidopsis*: CONSTANS is a hub for signal integration. Plant Physiol..

[15] Corbesier L., Vincent C., Jang S., Fornara F., Fan Q., Searle I., Giakountis A., Farrona S., Gissot L., Turnbull C. (2007). FT protein movement contributes to long-distance signaling in floral induction of *Arabidopsis*. Science.

[16] Sun R., Ding Y., Mimura M., Nishide N., Izawa T. (2025). Temporal transcriptome analysis reveals the two-phase action of florigens in rice flowering. Theor. Appl. Genet..

[17] Ding W., Gou Y., Li Y., Li J., Fang Y., Liu X., Zhu X., Ye R., Heng Y., Wang H. (2024). A jasmonate-mediated regulatory network modulates diurnal floret opening time in rice. New Phytol..

[18] Gou Y., Heng Y., Ding W., Xu C., Tan Q., Li Y., Fang Y., Li X., Zhou D., Zhu X. (2024). Natural variation in *OsMYB8* confers diurnal floret opening time divergence between *indica* and *japonica* subspecies. Nat. Commun..

[19] Cai X., Zeng X., Wang X., Pan D., Zhang J., Li Z., Yang J., Zhang Y., Zeng J., Zhang Q. (2025). Hormone metabolic profiling and transcriptome analysis reveal phytohormone crosstalk and the role of *OfERF017* in the flowering and senescence of sweet osmanthus. Hortic. Plant J..

[20] Zhang Y., Yang J., Zeng X., Cai X., Li Z., Zeng J., Zhang Q., Chen H., Zou J. (2025). Exploring miRNA–target modules regulating flower opening and senescence in *Osmanthus fragrans* through integrated transcriptome, miRNAome, and degradome analysis. Ind. Crops Prod..

[21] Wang J., Zhou Y., Zhang M., Li X., Liu T., Liu Y., Xie H., Wang K., Li P., Xu Z. (2025). Resolving floral development dynamics using genome and single-cell temporal transcriptome of *Dendrobium devonianum*. Plant Biotechnol. J..

[22] Chen X., Sun S., Han X., Li C., Wang F., Nie B., Hou Z., Yang S., Ji J., Li G. (2023). Multiomics comparison among populations of three plant sources of Amomi Fructus. Hortic. Res..

[23] Guo Y., Li Y., Zhang P., Luo Z., Yin J., Ma X., Yuan C. (2025). Biosynthesis of camphane volatile terpenes in *Amomum villosum* Lour.: Involved genes and enzymes. Plants.

[24] Zhu Y., Li S., Zhao H., Li Q., Wang Y., Yang C., Li G., Wang Y., Zhang L. (2025). Transcriptome sequencing-based analysis of premature fruiting in *Amomum villosum* Lour. Biology.

[25] Wang D., Zhu Y., Li S., Zhao H., Wang C., Li Q., Wang Y., Yang C., Li G., Wang Y. (2025). Cloning, expression, and bioinformatics analysis of the *AvFD1* gene in *Amomum villosum* Lour. Biology.

[26] Wang D., Wang C., Li S., Zhu Y., Li Q., Zhang W., Zhao H., Wang Y., Zhang L. (2025). Cloning and expression analysis of the *Ghd7* gene regulated by anaphase in *Amomum villosum* Lour. Trop. Agric. Sci. Technol..

[27] Smaczniak C., Immink R.G., Angenent G.C., Kaufmann K. (2012). Developmental and evolutionary diversity of plant MADS-domain factors: Insights from recent studies. Development.

[28] Stewart D., Graciet E., Wellmer F. (2016). Molecular and regulatory mechanisms controlling floral organ development. FEBS J..

[29] Liu L., Liu C., Hou X., Xi W., Shen L., Tao Z., Wang Y., Yu H. (2017). FTIP1 is an essential regulator required for florigen transport. PLoS Biol..

[30] Zhu Y., Liu L., Shen L., Yu H. (2016). *NaKR1* regulates long-distance movement of *FLOWERING LOCUS T* in *Arabidopsis*. Nat. Plants.

[31] Takagi H., Lee N., Hempton A.K., Purushwani S., Notaguchi M., Yamauchi K., Shirai K., Kawakatsu Y., Uehara S., Albers W.G. (2025). Florigen-producing cells express FPF1-LIKE PROTEIN 1 to accelerate flowering and stem growth in *Arabidopsis*. Dev. Cell.

[32] Fang K., Liu Y., Wang Z., Zhang X., Zou X., Liu F., Wang Z. (2023). Genome-wide analysis of the CaYABBY family in pepper and functional identification of *CaYABBY5* in the regulation of floral determinacy and fruit morphogenesis. Hortic. Res..

[33] Lin T., Yuan C., Dong C., Zeng M.G., Yang Y., Mao Z.C., Lin C. (2024). Screening and functional analysis of gene *CqSTK* associated with gametophyte development of Quinoa. Biotechnol. Bull..

[34] Tang N., Wu P., Cao Z.Y., Liu Y.N., Zhang X., Lou J., Liu X., Hu Y., Sun X.F., Wang Q.Y. (2023). A NAC transcription factor *ZaNAC93* confers floral initiation, fruit development, and prickle formation in *Zanthoxylum armatum*. Plant Physiol. Biochem..

[35] He Z.Y., Wu X.Y., Zhou W., Lei D., Yang J., Zou J. (2021). Cloning and expression analysis of the MADS-box family gene *HaAGL11* in sunflower. J. Plant Physiol..

[36] Wen K.X., Liu X.M. (2010). The important role of AP2 functional genes in plant floral development. Biotechnol. Bull..

[37] Lu H., Deng Q., Wu M., Wang Z., Wei D., Wang H., Xiang H., Zhang H., Tang Q. (2021). Mechanisms of alternative splicing in regulating plant flowering: A review. Chin. J. Biotechnol..

[38] Posé D., Verhage L., Ott F., Yant L., Mathieu J., Angenent G.C., Immink R.G.H., Schmid M. (2013). Temperature-dependent regulation of flowering by antagonistic *FLM* variants. Nature.

[39] Kinmonth-Schultz H.A., Tong X., Lee J., Song Y.H., Ito S., Kim S.-H., Imaizumi T. (2016). Cool night-time temperatures induce the expression of *CONSTANS* and *FLOWERING LOCUS T* to regulate flowering in Arabidopsis thaliana. New Phytol..

[40] Martina B., Nadine D., Christian J. (2015). Flowering time regulation in crops—What did we learn from *Arabidopsis*?. Curr. Opin. Biotechnol..

